# Interactions between local medical systems and the biomedical system: a conceptual and methodological review in light of hybridization subprocesses

**DOI:** 10.1186/s13002-023-00637-w

**Published:** 2023-12-13

**Authors:** Marina Barros Abreu, Thais Samila de Oliveira Ferraz, Ulysses Paulino Albuquerque, Washington Soares Ferreira Júnior

**Affiliations:** 1 Programa de Pós-Graduação em Etnobiologia e Conservação da Natureza, Universidade Federal Rural de Pernambuco, Rua Dom Manuel de Medeiros, s/n - Dois Irmãos, Recife, PE 52171-900 Brazil; 2 Laboratório de Investigações Bioculturais no Semiárido, Universidade de Pernambuco, Campus Petrolina, Rodovia BR 203, Km2, s/n – Vila Eduardo, Petrolina, PE 56328903 Brazil; 3 Laboratório de Ecologia e Evolução de Sistemas Socioecológicos, Departamento de Botânica, Universidade Federal de Pernambuco, Recife, PE 50670-900 Brazil

**Keywords:** Ethnobiology, Intermedicality, Medicinal plants, Pharmaceuticals, Resilience

## Abstract

Local medical systems (LMSs) are complex and dynamic, encompassing local perceptions of diseases, prevention and treatment strategies, and evaluations of therapeutic responses. These systems are not isolated and interact with other medical systems, such as the biomedical system. The interaction between these systems creates a "contact zone", which some authors refer to as intermedicality, involving both competitive and complementary interactions. However, there is limited discussion in the literature regarding the complexity of these interactions. Some studies seek to understand this interaction through the lens of hybridization, a concept introduced to ethnobiology by Ana Ladio and Ulysses Albuquerque. The authors conceptualize hybridization as "discrete structures and practices coming together to form a new practice not necessarily implying homogenization." They discuss hybridization in the context of medicinal plants used in urban settings and propose seven hybridization subprocesses to gain a deeper understanding of this phenomenon. In this study, we update these hybridization subprocesses, expanding the concepts to comprehend the specific interaction of resources from LMS and biomedical systems known and used by different human groups. In this context, we propose a new subprocess and have made adjustments to the existing subprocesses to encompass the diversity of possible interactions between medicinal plants and pharmaceuticals, providing evidence from the literature demonstrating interactions that can be classified within the proposed subprocesses. Furthermore, we discuss, from a theoretical standpoint, how these subprocesses may have implications for the resilience of medical systems. Moreover, we propose a flowchart that can be utilized to identify these hybridization subprocesses in intermedicality contexts in future studies. These classifications are crucial because they enable us to comprehend the complexity of interactions between medicinal plants and pharmaceuticals, as well as the impacts that these different interactions can have on the resilience of LMSs.

**Supplementary Information:**

The online version contains supplementary material available at 10.1186/s13002-023-00637-w.

## Background

Local Medical Systems (LMSs) are biocultural constructions encompassing a body of knowledge and practices embedded within local strategies for addressing illnesses [49]. These strategies encompass the perception of symptoms and causes, utilization of various therapeutic approaches, and assessment of treatments [1, 2]. Due to being open systems, LMS do not exist in isolation and interact with other systems [49], such as the biomedical system [3]. Biomedicine, referred to as Western medicine or cosmopolitan medicine [4], represents a globally standardized and scientifically validated approach to managing diseases [5–7]. Numerous studies have explored the dynamics of interaction between LMS and the biomedical system within diverse sociocultural and environmental contexts [8–12]. However, researchers have employed different terminology to describe these interactions. For instance, Greene [50] employed the term "intermedicality" to denote the specific space created from the interaction between different medical systems [see also 11]. Meanwhile, other studies have used terms such as “Medical Pluralism” [8, 10, 13, 14] and “syncretism” [9, 10] to indicate such interactions. In another example, Gale [51] criticizes the implications of adopting different terms in various studies that have assessed interactions between medical practices and discusses the use of different concepts employed in studies, such as "pluralism," "incorporation," "integration," "hybridity," and "activism." Given the diversity of terms, we employ the term “intermedicality” throughout the text as it encompasses a broad range of interactions arising from the contact of the two systems, including both complementarity and competition. In this study, we consider complementarity interactions as those wherein the practices of both systems aid individuals in coping with illness, thereby rendering the systems nonmutually exclusive. On the other hand, competitive interactions refer to instances wherein one system excludes the other.

When considering complementary interactions between systems, biomedicine can assist human groups in managing disease situations by providing an increase in the number of therapeutic responses adopted by people [10]. This can be observed, for instance, when pharmaceuticals are indicated for diseases where traditional or local strategies are not known [12], thereby expanding the range of treatment options individuals must consider when dealing with diseases. Conversely, in certain contexts, the presence of one system can prevent the knowledge and/or utilization of another system, giving rise to competitive interactions between them. For example, Vandebroek et al. [15] observed a negative relationship between the use of pharmaceutical products and the number of medicinal plants cited within indigenous communities in Bolivia. This evidence suggests that the use of pharmaceuticals may be displacing knowledge of medicinal plants within the communities under study. While various studies have been conducted to investigate the different uses of medicinal plants and pharmaceuticals among various human groups, addressing both competitive and complementary contexts, there is still a need to conduct research that can systematize the wide array of interactions between these elements in human populations. This can guide future studies and promote a deeper understanding of the complexity of these interactions.

The range of interactions between biomedicine and different local medical systems, whether complementary or competitive, can be interpreted from a broader perspective by considering the concept of hybridization proposed by Ladio and Albuquerque [16]. Hybridization represents a "dynamic process in which patterns can be identified both in space and time," becoming evident when diverse and distinct systems "coexist in the same space" [16]. In this context, hybridization can be observed through the (harmonious or not) coexistence of various cultures in relation to dietary, religious, and even musical practices [17]. However, within the scope of this article, the approach to hybridization aims to specifically understand the interaction between different medical systems, which we refer to as intermedicality. The authors put forward seven hybridization subprocesses to specifically understand the use of medicinal plants in urban settings: (1) fusion or juxtaposition, (2) recombination, (3) relocalization, (4) restructuring, (5) new developments in production, circulation, and consumption, (6) simultaneous coexistence of different symbolic universes and (7) spatial segregation. This perspective is interesting as it "*includes quali-quantitative aspects that can be studied in a concrete way in cities*” [16, p.7]. Furthermore, it provides insights into detecting patterns in the dynamic process of interaction between different medical systems.

In this study, we build upon the authors' proposal and suggest an updated set of hybridization subprocesses, expanding their applicability to nonurban contexts. While urban environments allow for interactions between different systems that align with the processes developed by Ladio and Albuquerque [16], a challenge arises in assessing the adaptation of these subprocesses to nonurbanized settings, necessitating the inclusion of new subprocesses. These updates are proposed due to the absence, to the best of our knowledge, of studies in the literature addressing the diversity of interactions that may emerge from the contact between biomedicine and local medical systems (LMS). Understanding the types of interactions, whether complementary or competitive, can provide insights into individual and collective adaptive mechanisms underlying the treatment choices by human groups in disease situations.

From the perspective of hybridization, we will examine the interactions that can arise from the contact between biomedicine and local medical systems in the following sections. We propose an expansion of the hybridization subprocesses, supported by evidence from the literature, and suggest a flowchart for systematically identifying these subprocesses. Additionally, we propose questions that can be addressed in future studies.

Here, two highly relevant points deserve attention. Firstly, we do not consider medicinal plants and pharmaceuticals (produced and marketed by industries and pharmacies, e.g. pills, syrups, vaccines, etc.) as necessarily opposing elements. We understand them as therapeutic strategies that can be found in different human groups around the world. Some countries have incorporated medicinal plants into their official healthcare systems, as is the case in Brazil by including medicinal plants of interest in the public healthcare system [43]. However, pharmaceuticals can differ from medicinal plants, as the latter represent essential components in the construction of medical systems throughout human evolutionary history [44–46] and continue to be the only therapeutic option available to many groups without access to biomedical care. Therefore, medicinal plants represent a human biocultural construct for dealing with disease events in various local medical systems.

On the other hand, pharmaceuticals have a more recent history and have become increasingly common worldwide. Regardless of whether medicinal plants are integrated into a country's official medicine or not, it is a fact that local medical systems have interacted and incorporated pharmaceuticals into their local practices, just as urban populations have employed medicinal plants. This suggests that medicinal plants and pharmaceuticals interact in diverse ways, and it is important to investigate how these elements interact and how this affects the dynamics and evolution of medical systems in various human groups over time.

The second point to consider is that various interactions can also be observed among medicinal plants, as different local or traditional medical systems can hybridize. For example, a set of studies has investigated how human migrations affect the knowledge and use of medicinal plants, considering processes of plant substitution in the new environment, for instance [47], which may be relevant for understanding the hybridization of local medical systems. This likely occurred significantly during human evolution. Although this is quite interesting, we will focus solely on the interactions between medicinal plants and biomedical elements, exploring the hybridization subprocesses linked to knowledge, practices, and beliefs that may reflect the interactions between these components. We will not delve into the specifics of hybridization subprocesses that may occur exclusively among medicinal plants within LMS.

## Interactions between medicinal plants and biomedicine in the light of hybridization subprocesses

In this section, we examine intermedicality from a broader perspective: hybridization. This term was initially employed in the field of Social Sciences and was defined by Canclini [17] as "sociocultural processes in which discrete structures or practices, which existed separately, combine to generate new structures, objects and practices". In the context of medical systems, hybridization can involve combining elements from both biomedicine and LMSs for the treatment of diseases. However, Canclini [17] suggests that these "discrete structures and practices" are not pure sources themselves, as they are the result of previous hybridizations. For instance, local pharmacopoeias are not static and may change, such as the incorporation of exotic species [11], implying that local medical systems may be a product of multiple hybridizations occurring over time.

Additionally, Canclini [17] proposes a focus on “hybridization processes” rather than solely on the integration of cultures, acknowledging the contradictions and elements that do not mix. Ladio and Albuquerque [16] adopt the term “hybridization” to highlight various subprocesses in the use of medicinal plants within urban environments, referring to the coexistence of different systems in the same space without necessarily leading to blending or homogenization between them. For example, when discussing hybridization in intermedicality scenarios, we also encompass situations where individuals substitute one system for another, among other instances of competition between systems.

In this context, Ladio and Albuquerque [16] proposed the aforementioned seven subprocesses to base ethnobiological research on medicinal plants in urban areas. A concise description for each subprocess suggested by the authors can be found in Additional file 1. However, upon examining evidence from other studies, particularly regarding the utilization of medicinal plants and pharmaceuticals within different human groups, we have identified situations that are not encompassed by the subprocesses delineated by Ladio and Albuquerque [16]. To identify these studies, we conducted a systematic review to assess the diversity of interactions between medicinal plants and pharmaceuticals in different human groups. This systematic review will be detailed in another manuscript currently in preparation and involved the search for literature in the Web of Science, Scopus, Scielo, and PubMed databases. We employed combinations of keywords representing medicinal plants and traditional medical systems ("medicinal plants," "traditional medical system," "local medical system," "traditional remedies"), along with terms related to biomedicine and pharmaceutical drugs ("biomedicine," "western medicine," "allopathic"). Upon examining the studies identified in the search, we indicate the need for adjustments to existing subprocesses and the incorporation of new ones. For instance, certain studies demonstrate that in intermedicality scenarios, people may initially turn to one treatment system (utilizing plants), and if symptoms do not improve, they seek another system (biomedicine) [3, 18]. In this regard, in certain situations, individuals may follow a specific order in seeking treatments, encompassing different systems throughout the course of the illness, which Schwartz [52] referred to as a "hierarchy of resort." In studies conducted within the Manus culture in the Admiralty Islands, Schwartz [52] identified two main sequences of treatment for illnesses. One involves an acculturative sequence, where allopathic medicine and the use of pharmaceuticals are the initial choices, followed by local and traditional strategies if the initial approaches prove ineffective. The other is the counter-acculturative sequence, in which traditional strategies are chosen initially, shifting to biomedical strategies if symptoms persist. The adoption of these sequences can be influenced by various factors, ranging from confidence in a specific medical system to the perception of the severity of illness symptoms [53]. There are also instances where pharmaceuticals are indicated for the treatment of diseases that cannot be addressed by medicinal plants [12], suggesting that the biomedical system can fill therapeutic gaps within the local medical system. Consequently, we propose adaptations to the subprocesses to encompass the diversity of interactions between medicinal plants and pharmaceuticals among various human groups in future investigations. In Additional file 1, we present the updates we have made to the subprocesses, accompanied by a brief explanation. In the subsequent paragraphs, we will elaborate further on the adaptations we propose. All subprocesses highlighted in this article can be observed at an individual level, except for one subprocess (segregation), which can only be observed at a community level.

### Subprocesses that can be assessed at the individual level

All hybridization subprocesses (Additional file 1) can be understood within the logic of complementarity or competition between the systems. In the scenario of complementarity, it is possible to frame the subprocesses of fusion, recombination, relocalization, innovations, and simultaneous coexistence of different symbolic universes. The first updated subprocess is the "fusion" subprocess. Ladio and Albuquerque [16] propose that fusion can be observed *"…when, in the cities, different species and practices are added, increasing the total richness of medicinal plants.*" [16, p. 4]. We suggest that, in intermedicality scenarios, fusion can occur through two pathways: fusion—diversification and fusion—sequential use.

#### Fusion: diversification

**Fig. 1:**
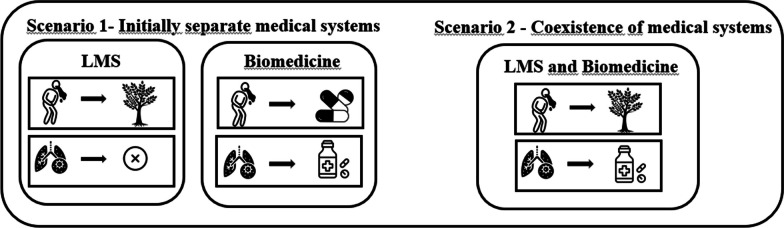
Fusion—diversification—Scenario 2: In the first scenario, medical systems are analyzed separately. The local medical system addresses vomiting but lacks a therapeutic strategy for respiratory diseases, whereas the biomedical system covers both areas. In the second scenario, fusion-diversification occurs, where, in the coexistence of both systems, the local strategy is retained for vomiting, and the biomedical system is integrated to address respiratory diseases. Thus, the biomedical system complements the local one, filling therapeutic gaps and diversifying the system, aligning with the Diversification Hypothesis, hence the term "Fusion-Diversification”

Fusion diversification can be observed when pharmaceuticals are used by a human group to address therapeutic gaps not covered by local medicine (medicinal plants). For instance, when exclusive use of biomedicine occurs to treat diseases that medicinal plants are ineffective against, or vice versa. In such cases, the coexistence of biomedicine with the local medical system (LMS) expands the range of responses available to deal with diseases (Fig. 1).

Regarding fusion, Ladio and Albuquerque [16] indicate that this subprocess suggests an increase in the total richness of strategies employed in the treatment of diseases. Based on the interactions between medicinal plants and pharmaceuticals in different human groups, we suggest that fusion can occur in two main ways. The first form can be discussed in the context of the diversification hypothesis proposed in the field of ethnobiology by Albuquerque [19]. This hypothesis suggests that exotic species are included in local pharmacopoeias to fill treatment gaps not addressed by native species [14, 20, 21]. The inclusion of pharmaceuticals in local medical systems can occur similarly to what the diversification hypothesis proposes. In this case, biomedical medicines can be incorporated into local medical systems to fill therapeutic gaps that medicinal plants do not fulfill and, consequently, generate an increase in the overall repertoire of medicines known/used by the community (fusion). In this work, we call this type of fusion "diversification". An example of this type of fusion can be found in Zank and Hanazaki [12], in a study conducted in Ceará and Santa Catarina, in the northeastern and southern regions of Brazil. The authors observed that medicinal plants are generally used to treat certain diseases, such as gastrointestinal problems, flu, and colds, while biomedicine is used for blood pressure problems and endocrine and nutritional diseases. This may suggest that the incorporation of pharmaceuticals may have occurred to fill therapeutic gaps left by medicinal plants. However, this differential use of plants and industrialized medicine may be a result of competitive interactions between systems, whereby pharmaceuticals can replace plants in certain diseases over time. If this occurs, the fusion subprocess is no longer applied. We will discuss the competition subprocesses later to further elaborate on these aspects.

#### Fusion: sequential use

**Fig. 2:**
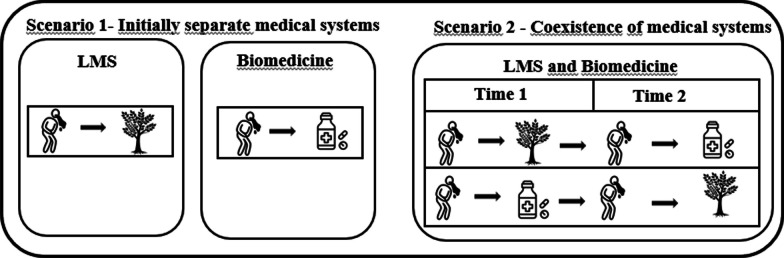
Fusion—sequential use—Scenario 2: In fusion—sequential use, the biomedical system complements the local medical system, expanding therapeutic options. Complementarity unfolds sequentially over time, observed in the evolution of treatment strategies. In the first scenario without hybridization, the local system treats vomiting with medicinal plants, while the biomedical system uses medications. The second scenario, involving hybridization, illustrates sequential use, where the initial treatment (Time 1) is with one system, and if there is no improvement, it transitions to the other (Time 2)

Fusion—sequential use can be observed when sequential use occurs, wherein one system is predominantly employed initially, and as the disease progresses, a transition is made to other system. This also leads to an increased diversity of responses, albeit sequentially based on the disease’s progression (Fig. 2).

Another form of fusion may involve cases where people initially resort to one treatment system (e.g., traditional) and, if they do not notice an improvement in the disease, seek the other system (e.g., biomedical) [8, 14]. This situation also reflects a diversity of possible responses for the treatment of one or several diseases and can be framed within the fusion subprocess but does not involve the use of biomedicine for specific diseases other than those for which plants are indicated. Therefore, we propose a second type of fusion subprocess, which we refer to as “sequential use”. For example, the study by Dræbel and Kueil [3] showed that people initiate treatment with medicinal plants from the local system, but as the disease is perceived as serious, they begin using pharmaceuticals. Evidence of sequential use can also be found when people initially resort to the cosmopolitan health system and later turn to the LMS. This was reported by Diaz et al. [18] when verifying that women in situations of terminal cancer, not cured by biomedicine, turned to the traditional medical system in search of healing through prayers and medicinal plants. These two possibilities are evidenced in the study by Odonne et al. [48], which investigated malaria treatments among the residents of Saint-Georges de l'Oyapock Village in French Guiana. In the study, 40.5% of individuals opted for herbal medicines when pharmaceutical drugs were not considered effective, while 20.4% began using pharmaceutical drugs when they realized herbal medicines were not working.

When considering the proposed fusion subprocesses, we understand that the "fusion-diversification" and "fusion-sequential use" subprocesses can arise from distinct mechanisms within a medical system. The "fusion-diversification" subprocess occurs when the incorporation process is employed to address therapeutic gaps within the system, akin to the introduction of exotic plants, representing a strategy within the medical system to fill the deficiencies left by native plants. This appears distinct from what we perceive as another aspect of the system related to "differential use," where certain elements, once incorporated into the system, may be prioritized over others due to various factors. In this context, the "fusion-sequential use" subprocess occurs through a "differential use" process, wherein, due to the progression of the disease, individuals may choose to discontinue the use of a medicinal plant in favor of other strategies, such as biomedicine. Therefore, the "fusion-diversification" subprocess is related to a broader process of incorporating elements into medical systems, while the "fusion-sequential use" subprocess is linked to something more specific, involving the mechanisms that regulate changes in the prioritization of strategies in specific disease episodes.

#### Recombination

**Fig. 3:**
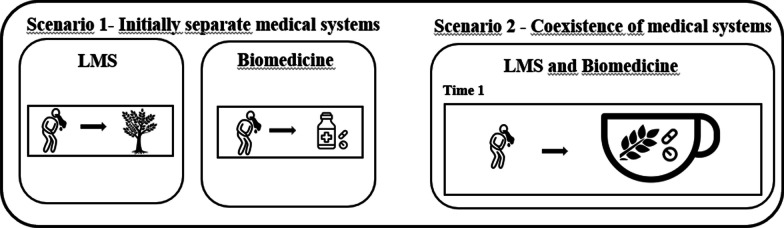
Recombination—Scenario 2: In scenario 2, recombination occurs when, in the coexistence of the LMS with the biomedical system, for the same health event, a person simultaneously uses elements from both the LMS and the biomedical system. The objective of this mixture can be to improve organoleptic properties or enhance therapeutic effects, among other benefits

Another hybridization subprocess proposed by Ladio and Albuquerque [16] is recombination, which would be observed “*when traditional and new elements mix, with the objective of increasing therapeutic action or improving the organoleptic properties of the preparation, without generating replacement*” [16, p. 4]. We propose that, in the intermedicality scenario, we can observe recombination when a combination of medicinal plants and pharmaceuticals is used together (mixtures) for the same disease (Fig. 3).

The concurrent utilization of pharmaceuticals and medicinal plants can be observed when there is a combination of medicines from both systems to enhance effectiveness and ensure a cure [7], which is considered recombination. In recombination, elements from both systems are simultaneously employed in the treatment. Ali-Shtayeh et al. [22] provide an example of this when they note that all patients receiving pharmaceuticals were also undergoing medicinal plants to delay disease progression, alleviate symptoms, and minimize side effects. Similarly, Tsimane women in the Amazon region combined traditional medicines with biomedicine in 13% of treatments, while men utilized this combination in 20% of treatments [42].

#### Relocalization

**Fig. 4:**
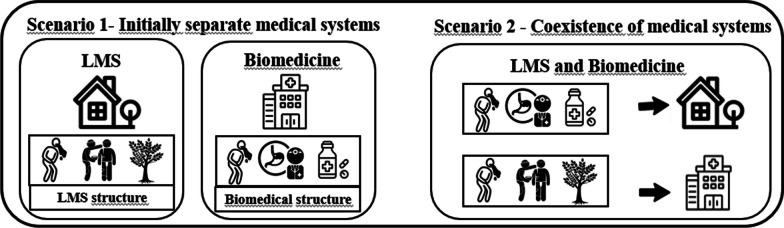
Relocalization—Scenario 2: In scenario 1, the systems are examined separately. In the biomedical system, the original structure consists of disease, healthcare professionals, and pharmaceutical medications in hospitals. In the LMS, the original structure comprises disease, local healers, and medicinal plants in local communities. To treat vomiting in the LMS, medicinal plants are used, while in the biomedical system, pharmaceutical medications are employed. In scenario 2, relocation can be observed, occurring when there is a change only in the physical environment while the structure of the medical system remains intact. This may involve biomedical professionals diagnosing and prescribing medications in local or traditional communities, as well as local healers diagnosing and treating diseases using the LMS within hospitals

Ladio and Albuquerque propose that relocalization occurs “*when resources and/or practices are reused or practiced in new physical ambits where they had not previously existed*” [16, p. 4]. We propose that, in the intermedicality scenario, if we consider that the basic structure of a given medical system is composed of the following components: “caregiver—therapeutic strategy—therapeutic target or disease” (e.g. a biomedical professional prescribing an industrialized medicine for treating influenza), relocalization occurs when the components of one medical system are employed in physical spaces that belong to another system (new physical context), without modifying the structure of the original system. For example, when biomedical professionals visit local communities to apply pharmaceuticals. In this case, the new physical context is the local community, as biomedical professionals operate within the physical spaces of biomedicine (hospitals, clinics, e.g.). This subprocess does not involve situations where people from the local community autonomously incorporate elements of biomedicine into their system; it solely pertains to the entry of biomedical professionals into the physical spaces of LMSs, bringing their knowledge and practices. The opposite can also occur when local or traditional specialists provide care within the physical spaces of biomedicine, such as hospitals (see 51 for situations in which doctors collaborate with traditional specialists) (Fig. 4). In some countries, traditional healthcare systems are institutionalized, and it is possible for local specialists to employ their traditional practices for healing patients in the physical settings belonging to biomedicine. This subprocess also does not cover situations in which biomedical professionals recommend traditional treatments during hospital appointments, as such cases involve a modification in one of the elements of the local or traditional system linked to the caregiver.

In certain instances, the components of a medical system are employed in new physical contexts, indicating the subprocess of relocalization. The relocalization subprocess is observed in a study conducted by Fontão and Pereira [23]. This research demonstrated that 12.1% of the consultations of the medical team of the “Mais Médicos” Program took place in community spaces within the village, allowing people to employ different systems (biomedical and traditional) as therapeutic strategies. This evidence suggests that biomedical professionals can utilize biomedical therapeutic strategies within the community, demonstrating that the components comprising the structure of the biomedical system (doctor + biomedical therapeutic strategies) remained unchanged, with only a shift in geographic space.

#### New developments in production, circulation, and consumption (Innovations)

**Fig. 5:**
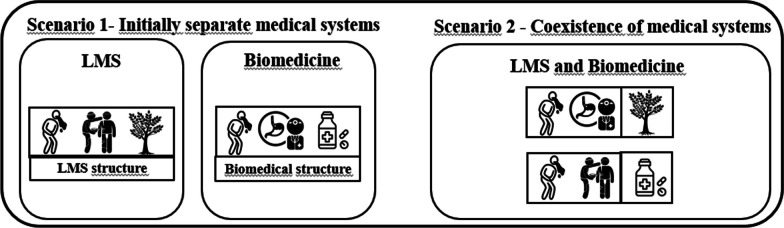
New developments in production, circulation, and consumption (Innovations): In scenario 1, the systems are analyzed separately. In the biomedical system, the original structure involves disease, healthcare professionals, and pharmaceutical drugs in hospitals. In the LMS, the original structure includes disease, local healers/self-treatment, and medicinal plants in local communities. In scenario 2, we observe innovations in the medical system structure in different physical spaces, such as local healers diagnosing and recommending medications in communities, and biomedical professionals using traditional strategies in hospitals

Ladio and Albuquerque propose that new developments in production, circulation, and consumption occurs “*when there are innovations in local therapies and their forms of acquisition, access and utilization*” [16, p. 4]. We propose that, in the intermedicality scenario, when any component of the structure of a given medical system undergoes modification within a hybridization context. For example, when pharmaceuticals are employed in new contexts, particularly local or traditional ones, by people from the traditional system. This can be observed when local healers begin using medical equipment (e.g., thermometers, oximeters) to aid in disease identification. Another interesting example is the use of pharmaceutical names for medicinal plants by local residents, such as “Novalgin”. In the context of biomedicine, this subprocess can also be observed when healthcare professionals recommend elements of local or traditional medicine to their patients (Fig. 5).

There are situations in which the interaction between systems can lead to structural changes in one of the systems when they are employed in new contexts. These changing situations suggest the subprocess of new developments in production, circulation, and consumption [14, 24]. The text by Whyte et al. [54] reflects on the sociology of medical practices in different historical periods, revealing that medicinal practices have migrated across continents, as evidenced in the exploration of the New World, where the use of plants by indigenous people spread to Europe. In this context, the dissemination of pharmaceutical products since the twentieth century represents another milestone in history, where various human groups have incorporated these products into their local practices. For instance, Gale [51] discusses a set of studies addressing the use of traditional medical practices by doctors, nurses, and pharmacists in their day-to-day activities in different regions. In another interesting example, the study by Medeiros et al. [14] demonstrated that people's visit to a local health center favored the choice of local treatments over biomedicine. According to the authors, this may have occurred due to the presence of health professionals who encourage the use of local or traditional treatments. Consequently, one of the fundamental components forming the structure of the local system has been modified, as local therapeutic strategies are indicated by biomedical professionals.

#### Simultaneous coexistence of different symbolic universes

**Fig. 6:**
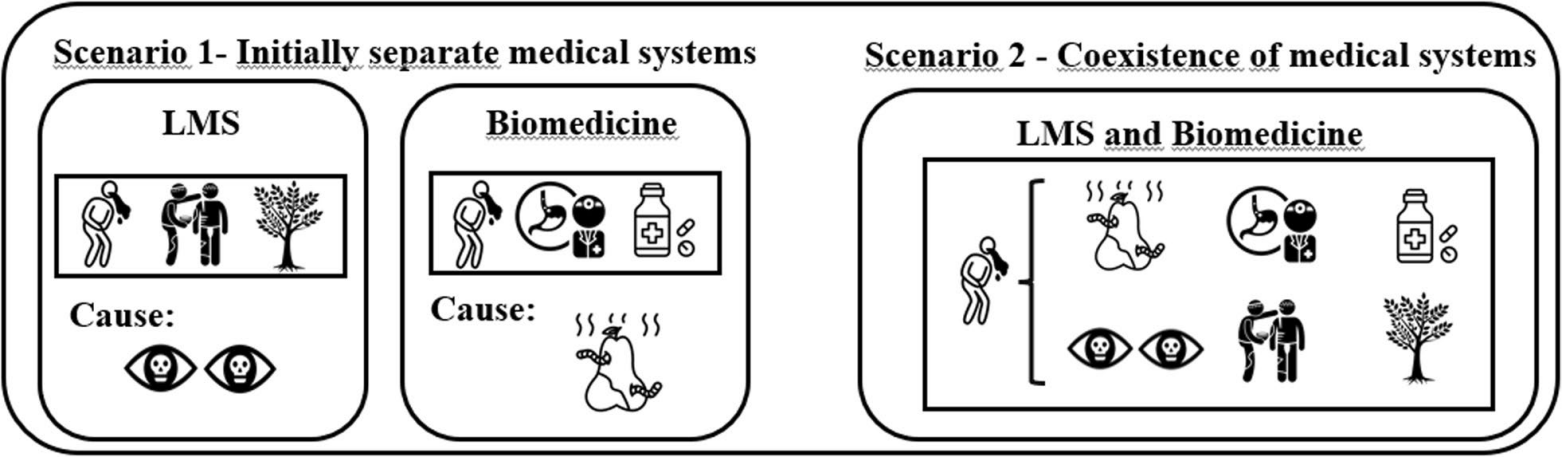
Simultaneous coexistence of different symbolic universes—Scenario 2: In Scenario 1, we highlight the difference in the perception of the causes of illnesses. For instance, in the LMS, vomiting can be attributed to spiritual issues or the evil eye, while in the biomedical system, the cause may be linked to the consumption of spoiled food. In Scenario 2, we demonstrate the coexistence of these two symbolic universes, allowing an individual to understand that the same illness may have different causes. This leads to seeking the LMS for spiritual causes and the biomedical system for physical causes of the disease, aiming to achieve holistic healing

Ladio and Albuquerque propose that simultaneous coexistence of different symbolic universes occurs “*when there is evidence of the coming together of different ways of perceiving health and illness and the different treatment methods of different cultural patrimonies.*” [16, p. 4]. We propose that, in the intermedicality scenario, when the same disease is understood differently by each system, yet they are not mutually exclusive. For instance, people may resort to pharmaceuticals to address the physical symptoms of an illness and turn to prayers and/or rituals to deal with the “spiritual symptoms” of the same illness. Similarly, biomedical treatment may be employed for certain disease groups, while traditional practices are used for “spiritual” ailments (Fig. 6).

Particularly concerning the simultaneous coexistence of different symbolic universes, a major challenge lies in the difficulty of finding literature that allows for the detection of this subprocess, as it involves ideological and symbolic scenarios [16]. The authors argue that the various proposed subprocesses (expanded upon here) possess aspects tied to therapeutic practices (plants, pharmaceuticals), as well as norms, cosmovisions, and beliefs that may be associated with practices. Despite these challenges, there is evidence of the simultaneous coexistence of different symbolic universes, such as the work of [54], which highlights situations where individuals incorporate pharmaceutical remedies into their local logic of efficacy. For instance, Hindley et al. [25], when studying the attitude of healers in Tanzania toward dementia, observed that the local population attributes various causes to the disease, including biological causes such as aging, as well as causes involving sorcery and witchcraft. Different causes can influence caregivers to make treatment decisions, whether it involves using biomedicine, medicinal plants, or prayers.

An intriguing aspect concerns a set of illnesses known as "culture-bound syndromes," which are culturally restricted to specific human groups and may have spiritual causes, finding no correspondence in the biomedical system [13, 55, 56]. In a context of hybridization, Halberstein [57] investigated the epidemiological profile of Caribbean migrants living in Miami, revealing that some mentioned illnesses culturally associated with their regions of origin, classified as "culture-bound syndromes." These individuals indicated treating such illnesses using pharmaceutical drugs supplemented with medicinal plants.

In another interesting example, Mathez-Stiefel et al.'s study [13] was conducted in various rural Andean communities in Peru and Bolivia, which have varying levels of access to biomedical services. The research observed that culturally rooted illnesses of spiritual origin, such as "bad wind," "fright sickness," "lightning," and "witchcraft," are treated by traditional healers and traditional medicine, while biomedical services are recommended for the treatment of other ailments. This suggests that different symbolic universes can coexist in human groups, even in the presence of biomedicine.

#### Competition subprocesses: restructuring and structure maintenance

**Fig. 7:**
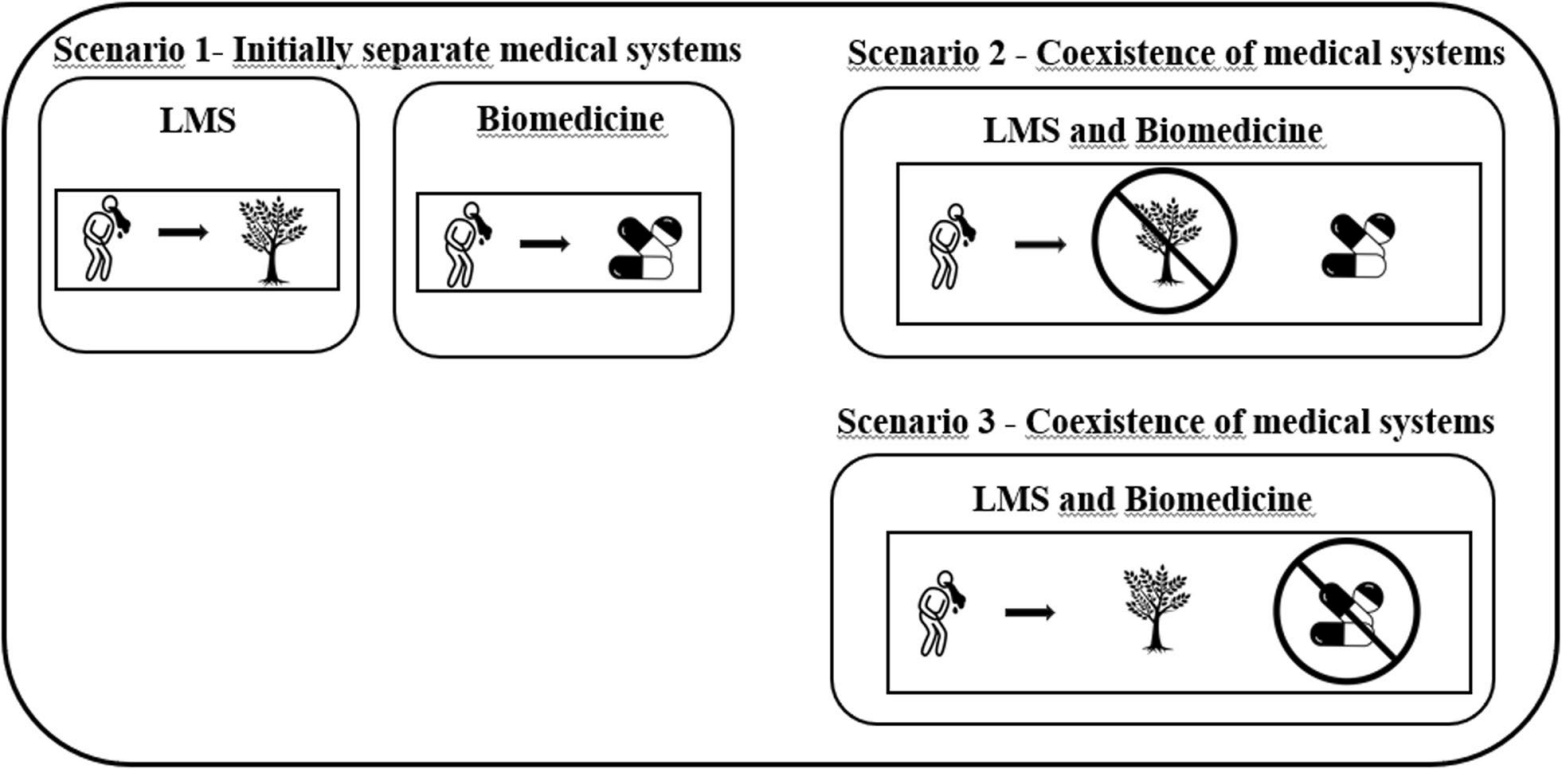
Restructuring—Scenario 2 and Structure maintenance—Scenario 3: In scenario 1, the systems are analyzed separately, where, for vomiting, in the LMS, people use medicinal plants, and in the biomedical system, they use pharmaceutical drugs. The restructuring is demonstrated in scenario 2, when both systems coexist in the same space, and people tend to prioritize the use of pharmaceuticals in disease treatment. The maintenance of the structure is demonstrated in scenario 3, when both systems coexist in the same space, and people tend to prioritize the use of medicinal plants in disease treatment

In addition to the scenario of complementarity, there are subprocesses that fall under competition (Fig. 7). In this scenario, two extreme situations can be observed. The first is when biomedicine poses a threat to LMS, which may lead to a decrease in the knowledge and use of medicinal plants by local populations, representing a restructuring subprocess (Fig. 7) [15]. Ladio and Albuquerque propose that restructuring occurs “*when changes and/or substitution of a resource are generated, due to scarcity or other factors, implying a significant change in the order of importance of the species used to treat a particular illness*” [16, p. 4]. We propose that, in the intermedicality scenario, when competition of biomedicine and LMS results in the substitution of local pharmacopeia for a particular disease or diseases in general. The introduction of biomedicine into local medical systems can lead to a decline in the knowledge or use of medicinal plants.

On the other hand, medical systems may respond differently to the presence of biomedicine, prioritizing the use of medicinal plants and impeding the spread of biomedicine within the community due to several factors (economic, cultural, among others) [26, 27]. For example, De Wet et al. [27] observed that most people in the studied community prefer the use of medicinal plants for skin conditions, even though more than 90% of the population has access to biomedicine through hospitals and clinics. Based on this evidence, we propose a new subprocess called "structure maintenance" (Fig. 7). This subprocess occurs when there is a prioritization of knowledge/use of medicinal plants over pharmaceutical drugs at the individual and/or collective level. This subprocess is the opposite of restructuring, and the competition between biomedicine and Local Medical Systems (LMSs) can lead to the prioritization of medicinal plants. In this scenario, LMSs tend to maintain their structure over time, favoring the individual elements within the system (medicinal plants) over biomedicine. This can occur due to factors such as a preference for medicinal plants, associated high costs, and perceived side effects of biomedicine, among others. While this subprocess reflects a competitive interaction, it can promote the structural resilience of LMSs, wherein the system retains its structure when dealing with disturbances. For instance, in a study with the Fulni-ô indigenous people, Soldati and Albuquerque [11] revealed that the community observed the increased adoption of nonindigenous elements among the younger generations. In response, the community initiated a movement to empower their traditional practices on multiple fronts, ensuring that their religious traditions (which involve medicinal plants and sacred plants) are not forgotten, despite the introduction of external elements such as biomedicine.

### Segregation: a subprocess that is only evaluated at the collective level in medical systems

**Fig. 8:**
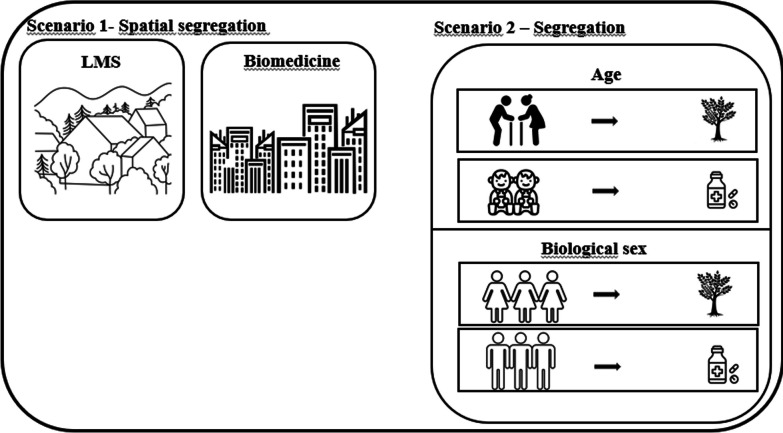
Segregation—Scenario 2: This specific subprocess is only observed on a group scale. In this case, other subprocesses can occur at the individual level and can be organized based on spatial groups, gender, age, among others. In Scenario 1, we present the original concept by Ladio and Albuquerque (2014), who focus primarily on spatial segregation, with groups forming in different geographical spaces. For example, rural communities tend to prioritize the LMS, while industrialized communities prioritize the biomedical system. In Scenario 2, we introduce the updates we propose, suggesting that groups can be formed based on age, gender, among other factors. For instance, there may be a tendency for the structure maintenance subprocess among females and the elderly, while a tendency for the restructuring subprocess can be observed among males and young individuals

Finally, we would like to emphasize the subprocess of segregation (Fig. 8). Ladio and Albuquerque propose that segregation occurs “*when internal groupings are formed in terms of species and practices that can be observed spatially, in the urban geography.*” [16, p. 4]*.* We propose that, in the intermedicality scenario, different groups within a community utilize the systems in different ways. These groups can be divided based on gender, age, occupation and spatially (Fig. 8). For example, women in a particular community may prefer medicinal plants, while men in the same community prefer pharmaceuticals. This subprocess exhibits intriguing aspects, as it can manifest as either a competition or a complementarity subprocess. For example, Singh et al. [28] examined the use of medicinal herbs for treating gynecological disorders in Tonga and observed that younger women in the community, closer to the urban center, solely relied on biomedicine (indicating a competitive interaction), while older women incorporated elements from both systems (complementarity). In this case, the subprocesses associated with segregation are connected to both competition and complementarity scenarios. This is because, while other subprocesses can also occur at an individual level, segregation can only be observed collectively on a population scale. Therefore, whenever there is segregation, other distinct subprocesses are simultaneously occurring at the individual level [29]. Such evidence aligns with Ladio and Albuquerque [16], who point out that the subprocesses are not mutually exclusive and can coexist in the same community.

#### Segregation

The subprocess of segregation can manifest in various ways, depending on the factors that shape the interactions between the systems at the individual level. For example, the study conducted by Alqethami et al. [30] in Jeddah, Saudi Arabia, provides evidence of competition by reporting that females exhibit a preference for using medicinal plants (structure maintenance), while males opt for biomedicine (restructuring). This demonstrates evidence of segregation based on gender. Another example, as previously mentioned by Singh et al. [28], reveals segregation based on age, as people from different age groups have preferences for treatments from different systems.

Given the diversity of hybridization subprocesses that can be evaluated at both the individual and collective levels, investigating the interactions between medical systems within human groups is a challenging task. To aid in identifying these subprocesses, we created a flowchart. Additional file 2 provides a more detailed visual representation, which can be utilized in future studies aiming to identify each subprocess within a group or across different human groups. This can facilitate standardized data collection in the identification of these subprocesses, enabling regional and global comparisons.

## Theoretical advances in hybridization research and their implications for LMS resilience

Taking into consideration the significant role that local medical systems play in disease management across various human groups, a crucial question arises regarding their ability to cope with disturbances that threaten their functions and processes over time [31, 32, 49]. Numerous studies have employed the concept of resilience to explore the capacity of these systems to endure adversity [33], investigating how they maintain their integrity when facing disturbances over time [33, 34]. Within ethnobiological studies, three interpretations of resilience have been observed: (1) structuralist, which focuses on structural changes within the system; (2) functionalist, which emphasizes system functions; and (3) processual, which concentrates on the processes governing system functions [33].

These three interpretations of resilience can be applied to local medical systems, and in each case, various factors can positively or negatively contribute to resilience. For example, the functional resilience of LMS, which pertains to the preservation of system functions (such as disease cure) in the face of disturbances (such as the local extinction of useful species or the introduction of biomedicine), can be enhanced through utilitarian redundancy [19, 35]. Utilitarian redundancy refers to a group of species that perform the same function, such as treating a particular disease within a local medical system [19]. Utilitarian redundancy is crucial because it can increase the system’s capacity to respond to disturbances (such as the local extinction of certain species). In the absence of a particular plant species, the system can adapt by utilizing other redundant species that were less frequently employed prior to the disturbance, thus preserving its therapeutic functions [19, 32].

Another factor influencing the resilience of LMS is its coexistence with biomedicine. Although several studies have examined this coexistence [7, 8, 12, 14], little attention has been given to the broader implications of these interactions. For instance, how do these interactions contribute to the resilience of local systems? [36]. Some authors argue that the interaction between biomedicine and LMS can have negative effects on the local system, suggesting that the arrival of biomedicine may lead to a decline in local knowledge or even its replacement [15, 30, 37, 38]. Other studies indicate a complementary relationship between the two systems, observing that the arrival of biomedicine does not necessarily result in a reduction in local knowledge, as knowledge about medicinal plants can increase alongside the knowledge and use of biomedicine among the population [10]. Alternatively, there may be a complementary utilization of both systems [7, 12]. However, the interactions between these systems are complex, and it is essential to conduct a specific assessment to determine the nature of the interaction and its implications for the different types of resilience.

From a structural perspective, the composition of medicinal plants known and used for disease treatment can be considered, as medicinal plants have been a very important component of various LMSs throughout human history [39]. If we view the arrival of biomedicine as a disturbance and assume that the interactions with the local system are complementary (fusion-diversification, fusion-sequential use, recombination), it does not necessarily impact the structural resilience of the LMS, as the medicinal plants being utilized are not lost (Table 1). However, if the LMS exhibits competitive interactions, such as restructuring, the structural resilience of the LMS would be negatively affected since biomedicine would alter the system’s structure by replacing local medicine. On the other hand, if there is a structure maintenance subprocess, the LMS would benefit positively. Even with the arrival of biomedicine, local strategies would be prioritized, thus preserving the structure.

**Table 1:** Hybridization subprocesses' impact on resilience types in the context of biomedicine as a disturbance

Disturbance: arrival of biomedicine					
Type of interaction	Complementarity			Competition	
Type of resilience/subprocess	Fusion—diversification	Fusion—sequential use	Recombination	Restructuring	Structure maintenance
Structuralist	0	0	0	–	+
Functionalist	0	0	0	–	+
Processual	±	±	±	±	±

“0”, No impact; "−", Negative impact; "+", Positive impact; "±", Impact can be positive or negative

In the functional interpretation applied to LMSs, it can be asserted that the resilient system is the one that continues to manage to treat diseases, even in the presence of structural changes (e.g., changes in plants and known practices). In this regard, when assessing the interaction between LMS and biomedicine, the subprocesses of complementarity follow a similar rationale to that observed in structural resilience. However, in cases of competition between the systems linked to the restructuring subprocess, if biomedicine completely replaces the LMS over time, it would be unable to maintain its functions, which would be entirely taken over by the biomedical system.

Different reasoning is used to understand processual resilience since a resilient LMS is one that not only fulfills its functions of managing disease situations but also maintains processes associated with disease management, such as treatment selection and knowledge transmission. Therefore, in this case, all types of interaction that can occur between biomedicine and LMS can have positive or negative outcomes, depending on whether the interaction is accompanied by the maintenance of the processes governing the LMS. For example, Etkin et al. [9] demonstrate biomedicine being incorporated within local processes (in the local logic of efficacy). Thus, in the processual logic, the system also displays resilience if biomedicine is integrated into LMS processes. In this scenario, even if biomedicine completely replaces local medicinal plants, the LMS remains resilient as long as it maintains the same local processes that were present with medicinal plants (e.g., the transmission of pharmaceuticals by local specialists; employing the logic of medicinal plant selection based on organoleptic properties, for example, being also used for pharmaceuticals) [33]. However, there is limited research with a processual focus, making it important to guide future investigations in this aspect.

## Future research to identify hybridization subprocesses in intermedicality

In the ethnobiological literature, there already exists a compilation of methods aimed at facilitating data collection in medical systems [40]. However, the selection of methods depends on the objective of each study, and in regard to identifying the aforementioned updated hybridization subprocesses, certain data that we observe to be absent in a set of ethnobiological works on which we rely to construct this article become crucial. Merely having a list of locally known and used plants and pharmaceuticals is insufficient for comprehending the interactions between these elements and the resulting consequences. In Table 2, we propose a series of questions that can be posed to grasp the complexity of interactions that medicinal plants and pharmaceuticals may exhibit, thereby guiding the development of research protocols.

**Table 2:** Questions that can guide the construction of methodological protocols in ethnobiological research within intermedicality scenarios

1	Which plants and pharmaceuticals are utilized for a specific disease?
2	How are these medications used? (This question can be complemented with questions provided in Box 6 of Ferreira Júnior et al. [40])
3	Is a system primarily used?
4	Is there a preference for any of the systems? Why?
5	Are there diseases that are exclusively treated using one of the systems? If so, what diseases are these? Why?
6	Are there diseases that have multiple treatments using both plants and medicines of biomedical origin? If so, what diseases are these? Why?
7	In cases where a specific system is used to treat a disease, is it due to the lack of alternatives from the other system, the progression of the disease with no observed cure, or another reason?
8	Does migration from one system to another occur during different stages of the disease?
9	Are there variations in treatments for chronic diseases?
10	Do different perceptions exist regarding the same disease?

The data needed to address the above questions can be obtained through individual interviews, such as employing free lists. These lists can be generated by asking participants to name known diseases or medicinal plants. Additional techniques, such as utilizing therapeutic itineraries, can be significant for gathering data on interactions between medical systems [40]. Therapeutic itineraries serve as a valuable complement to data collection, as they enable researchers to access information on the diseases experienced by people and how they were managed [40]. This is important to standardize data collection in future studies, enabling the comparison of various subprocess types in human groups across different scales in space and time. It allows for the assessment, at these scales, of factors that may influence the occurrence of different subprocesses.

In certain contexts, there is variation in the knowledge of medicinal plants and pharmaceuticals based on disease characteristics, such as frequency and severity. The incidence of a disease can be linked to a greater number of medicinal plants used and the sharing of information over time, as well as increased utilization of modern medicine [41]. In some cases, the combination of traditional and biomedical treatments can be attributed to the influence of frequency, severity, and chronic diseases together, rather than individual factors alone [7]. In a recent article, we emphasized the importance of comprehending the functionality of medical systems through the interaction of various factors, providing insights into the functional aspects of these systems, compared to the isolated analysis of these factors [49]. Such evidence highlights the importance of investigating various factors that may influence health-seeking behavior within a hybridization scenario in future studies. For instance, apart from the perceived characteristics of diseases, individuals’ perceptions of the local and biomedical systems can impact the interactions between medicinal plants and pharmaceuticals [8]. Hence, we propose important questions to understand the underlying phenomena behind hybridization subprocesses, which are outlined in Table 3.

**Table 3:** Important questions to understand the underlying phenomena behind hybridization subprocesses

1	Are the local medical system and the biomedical system considered distinct systems by the local population? If so, is there any hierarchy among these systems that is perceived by individuals?
2	Are there local healers within the community?
3	Who do people turn to as a reference when they need to treat diseases?
4	Do these practitioners work for free?
5	Do these practitioners dialogue with biomedical professionals?
6	How are traditional medicines obtained?
7	Is it necessary to buy medicinal plants?
8	What is the price of local remedies if they need to be bought?
9	How difficult is it to find local remedies in the surrounding vegetation?
10	How accessible is the cosmopolitan healthcare system?
11	How are biomedical health services accessed?
12	How is the dialogue between biomedicine and the local community?
13	Are healthcare services public or private?
14	If private, what is the value for consultation and remedies?
15	What is the form of acquisition of pharmaceuticals?

## Final considerations

This study aimed to propose theoretical and methodological frameworks for future research that explore the interaction of local medical systems (here in terms of medicinal plants) and biomedicine. The empirical evidence presented demonstrates that future investigations should go beyond mere lists of medicinal plants and pharmaceuticals that are known to treat diseases. It is crucial to comprehend how these plants and pharmaceuticals are used and how different medical systems are perceived by various communities. Furthermore, a theoretical effort is necessary to identify the factors that drive the adoption of different medical systems, allowing for a deeper understanding of the behaviors exhibited by human populations in their pursuit of health. It is impossible to conserve local medical systems without considering the positive or negative impacts that biomedicine has on local populations in an increasingly globalized world.

## Limitations

One limitation of this study is that it focuses on the hybridization subprocesses arising from the interactions between the local medical systems and the biomedical system. Medical systems are dynamic and complex and may have been the result of various hybridizations over time. To provide an example within local medical systems, it is challenging to assess the historical incorporation of species for medicinal use and to investigate how this incorporation may result from the interaction of different local medical systems [11]. In such cases, the presence of certain medicinal plants within a system may be the outcome of various hybridization subprocesses occurring between different LMSs over time. While this aspect is intriguing, we did not reflect the subprocesses of hybridization in these contexts, which could be the focus of future studies.

### Supplementary Information

**Additional file 1**: Description of the hybridization subprocesses and updates. Hybridization subprocesses and updates on interactions between medicinal plants and biomedical drugs in human groups.

**Additional file 2**: Subprocesses of hybridization - Flowchart. Flowchart to guide the identification of hybridization subprocesses between local medical systems and cosmopolitan medical system in different human groups.

## Data Availability

Not applicable.

## Abbreviations

LMS: Local medical systems
CMS: Cosmopolitan medical system
