# Supplementary material for: Interactions between local medical systems and the biomedical system: a conceptual and methodological review in light of hybridization subprocesses
Source: J Ethnobiol Ethnomed. 2023 Dec 13;19:60. doi: 10.1186/s13002-023-00637-w (PMC10720130; doi:10.1186/s13002-023-00637-w)
Supplement: Supplementary file 1 — Additional file 1: Description of the hybridization subprocesses and updates. Hybridization subprocesses and updates on interactions between medicinal plants and biomedical drugs in human groups. [file 13002_2023_637_MOESM1_ESM.pdf]

Additional file 1. Hybridization subprocesses and updates on interactions between medicinal plants and biomedical drugs in human groups.

Original concept by Ladio and Albuquerque [16]	Update of hybridization subprocesses in a context of intermedicality
**Fusion or juxtaposition:**<br><br>“*...when, in the cities, different species and practices are added, increasing the total richness of medicinal plants.*” [16, p. 4]	**Fusion - diversification:**<br><br>When pharmaceuticals are used by a human group to address therapeutic gaps not covered by local medicine (medicinal plants). For instance, when exclusive use of biomedicine occurs to treat diseases that medicinal plants are ineffective against, or vice versa. In such cases, the coexistence of biomedicine with the local medical system (LMS) expands the range of responses available to deal with diseases.<br><br>**Fusion - sequential use:**<br><br>When sequential use occurs, wherein one system is predominantly employed initially, and as the disease progresses, a transition is made to other system. This also leads to an increased diversity of responses, albeit sequentially based on the disease’s progression.
**Recombination**<br><br>“*when traditional and new elements mix, with the objective of increasing therapeutic action or improving the organoleptic properties of the preparation, without generating replacement.*” [16, p. 4]	**Recombination**<br><br>When a combination of medicinal plants and pharmaceuticals is used together (mixtures) for the same disease.
**Restructuring**<br><br>“*when changes and/or substitution of a resource are generated, due to scarcity or other factors, implying a significant change in the order of importance of the species used to treat*	**Restructuring**<br><br>When competition of biomedicine and LMS results in the substitution of local pharmacopeia for a particular disease or diseases in general. The introduction of biomedicine into local medical systems can

a particular illness." [16, p. 4]	lead to a decline in the knowledge or use of medicinal plants.
–	**Structure maintenance**<br><br>**(New subprocess)**<br><br>When there is a prioritization of knowledge/use of medicinal plants over pharmaceutical drugs at the individual and/or collective level. This subprocess is the opposite of restructuring, and the competition between biomedicine and Local Medical Systems (LMSs) can lead to the prioritization of medicinal plants. In this scenario, LMSs tend to maintain their structure over time, favoring the individual elements within the system (medicinal plants) over biomedicine.
**Relocalization**<br><br>"*when resources and/or practices are reused or practiced in new physical ambits where they had not previously existed*." [16, p. 4]	**Relocalization**<br><br>If we consider that the basic structure of a given medical system is composed of the following components: "caregiver - therapeutic strategy - therapeutic target or disease" (e.g. a biomedical professional prescribing an industrialized medicine for treating influenza), relocalization occurs when the components of one medical system are employed in physical spaces that belong to another system (new physical context), without modifying the structure of the original system. For example, when biomedical professionals visit local communities to apply pharmaceuticals. In this case, the new physical context is the local community, as biomedical professionals operate within the physical spaces of biomedicine (hospitals, clinics, e.g.). The opposite can also occur when local or traditional specialists provide care within the physical spaces of biomedicine, such as

	hospitals.
**New developments in production, circulation, and consumption**<br><br>"*when there are innovations in local therapies and their forms of acquisition, access and utilization*." [16, p. 4]	**New developments in production, circulation, and consumption (Innovations)**<br><br>When any component of the structure of a given medical system undergoes modification within a hybridization context. For example, when pharmaceuticals are employed in new contexts, particularly local or traditional ones, by people from the traditional system. This can be observed when local healers begin using medical equipment (e.g., thermometers, oximeters) to aid in disease identification. Another interesting example is the use of pharmaceutical names for medicinal plants by local residents, such as "Novalgin". In the context of biomedicine, this subprocess can also be observed when healthcare professionals recommend elements of local or traditional medicine to their patients.
**Simultaneous coexistence of different symbolic universes**<br><br>"*when there is evidence of the coming together of different ways of perceiving health and illness and the different treatment methods of different cultural patrimonies.*" [16, p. 4]	**Simultaneous coexistence of different symbolic universes**<br><br>When the same disease is understood differently by each system, yet they are not mutually exclusive. For instance, people may resort to pharmaceuticals to address the physical symptoms of an illness and turn to prayers and/or rituals to deal with the "spiritual symptoms" of the same illness. Similarly, biomedical treatment may be employed for certain disease groups, while traditional practices are used for "spiritual" ailments.

**Spatial segregation**<br><br>"*when internal groupings are formed in terms of species and practices that can be observed spatially, in the urban geography*." [16, p. 4]	**Segregation**<br><br>When different groups within a community utilize the systems in different ways. These groups can be divided based on gender, age, occupation and spatially. For example, women in a particular community may prefer medicinal plants, while men in the same community prefer pharmaceuticals.
