# Supplementary figures and images for: Interactions between local medical systems and the biomedical system: a conceptual and methodological review in light of hybridization subprocesses
Source: J Ethnobiol Ethnomed. 2023 Dec 13;19:60. doi: 10.1186/s13002-023-00637-w (PMC10720130; doi:10.1186/s13002-023-00637-w)

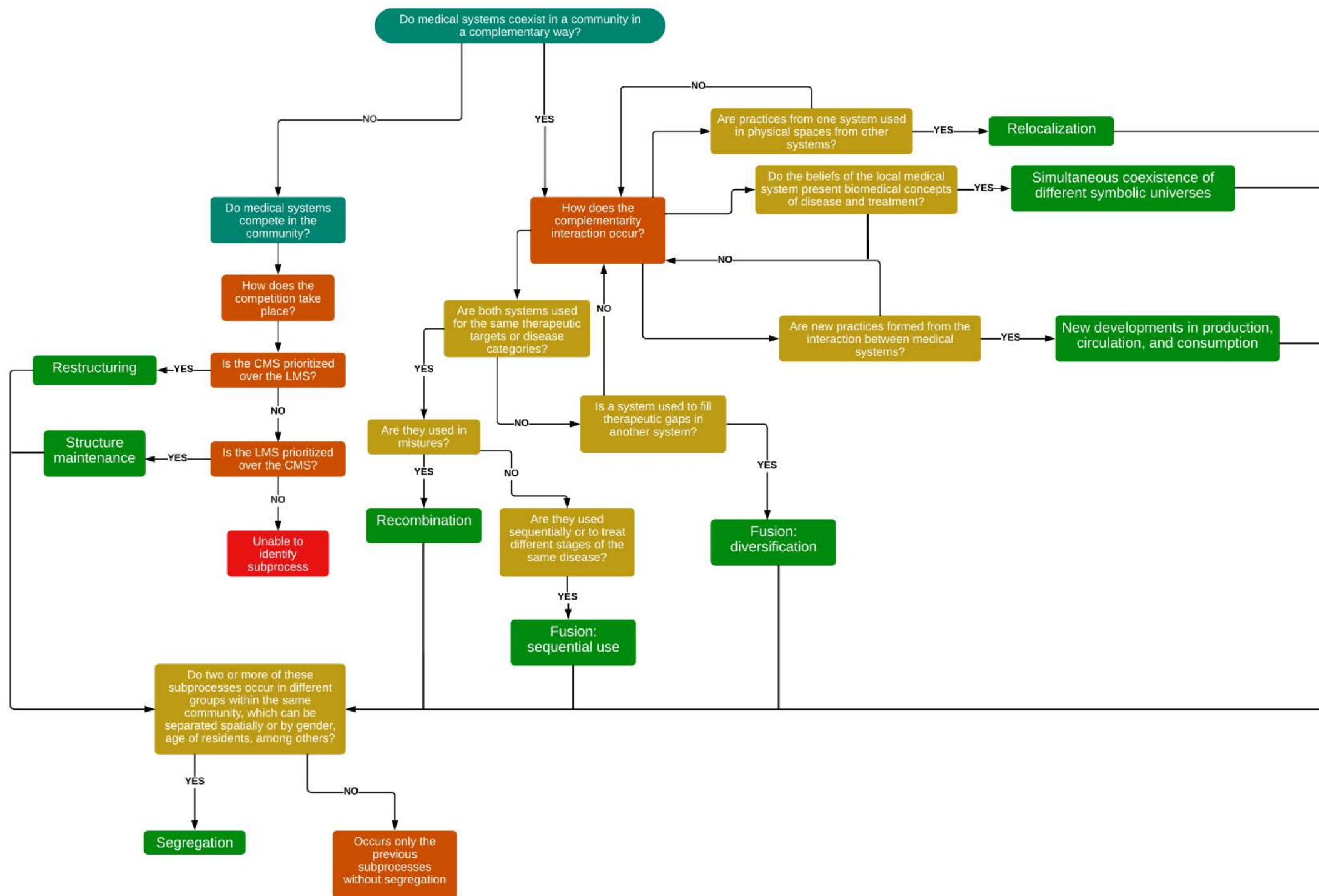

Supplement: Supplementary file 2 — Additional file 2: Subprocesses of hybridization - Flowchart. Flowchart to guide the identification of hybridization subprocesses between local medical systems and cosmopolitan medical system in different human groups. [file 13002_2023_637_MOESM2_ESM.pdf]
